# The Westminster Hospital

**Published:** 1895-07-13

**Authors:** 


### THE WESTMINSTER HOSPITAL.

The Westminster Hospital, as is shown by the figures contained in *Burdett's Hospital and Charities Annual*, 1895, is the most economically conducted hospital in the country. Despite the economy of the management, its efficiency is equal to that of any similar institution, and it undoubtedly deserves the cordial support of every intelligent Londoner who is interested in the maintenance of the voluntary system of hospitals. The authorities have wisely decided to make the festival dinner a real festival, by holding it at long intervals. The gathering on Saturday, the 6th inst., will be memorable from the circumstance that the most eloquent speeches were made by soldiers, although the speakers included Cardinal Vaughan, Viscount Knutsford, Mr. Burdett-Coutts, M.P., the Dean of Westminster, and others. Dr. Allchin made a statesman-like speech, in which he took up some of the most warmly contested points in modern hospital administration, and handled them with discretion and force. The Duke of Connaught justly commended Dr. Allchin's speech, which we shall hope to publish in full on another occasion. Lord Methuen's speech was one of the ablest we have ever heard from a soldier, and the Duke of Connaught surpassed himself on this occasion. The Duke has the gift of eloquence. He evidently speaks from his heart, and marshals his facts with convincing force. He reminded us very much of the late Duke of Albany, and we hope it may be possible for him to gradually take a more active part in public ceremonies of this kind than he can hope to do at present owir to the pressure of his military duties. He is entitled to the warm thanks of everybody connected with the Westminster Hospital, from the circumstance that he had to come to London twice and to return to Aldershot on Saturday to take part in a heavy field day and be present at the distribution of prizes before he presided at the dinner at the Whitehall Rooms at eight o'clock. We are pleased to note that although the total contributions in connection with the festival only reached a little over £4,000, this action of His Royal Highness, and the proud position which the Westminster Hospital occupies as the most economically administered in the country, led one of those present to undertake to raise the total sum to £5,000 before the end of this year. We were also glad that warm appreciation was manifested by those present at the dinner on Saturday for Mr. Sidney M. Quennell, the able and courteous secretary, whose great services entitle him to receive some substantial mark of appreciation at the hands of the Committee of Management.

Speech of H.R.H. the Duke of Connaught.

The Chairman, in proposing the toast of the evening, said that the Westminster Hospital, started in 1619, was one of the first charities of its kind that had been founded on the voluntary system. That system meant a great deal, and Englishmen had a right to be proud of the fact that great and important institutions, such as hospitals, were maintained purely by voluntary subscriptions. (Cheers.) He thought that in this respect we stood almost alone. Admirable, however, as the system was, its vicissitudes were only too evident to all. The Westminster Hospital had 205 beds, and every year relieved about 3,000 in-patients and 24,000 out-patients. This might be thought a small number in comparison with that recorded at other hospitals, but the figures obviously pointed to the alleviation of very much suffering. (Cheers.) The Westminster Hospital, while a general one, had special departments for the treatment of the diseases of women and children, and it was not so long ago that, in connection with the Queen's Jubilee, the Duchess of Connaught opened the children's ward now bearing her name. (Cheers.) Another important feature of the hospital was a training school for nurses. He could only express the feeling of all present when he said that they recognized the importance of having a medical school at the hospital. The experience gained by students at such a school was invaluable, not only to the metropolis of this country, but to humanity in general. He was well aware of the immense expense of maintaining the schools, but he thought that every effort should be made to maintain them—and he laid stress upon the point—because he was certain that the experience gained by young medical men was such as could not be gained elsewhere. (Cheers.) The result, therefore, was most important to the community at large. The expenditure for 1893 and 1894 had exceeded the receipts by £5,463 and £4,147 respectively. To supply the deficiency it became necessary to sell out £11,000 from the small reserve fund, so that the annual income was diminished by £330. By this means, however, the hospital avoided getting into debt with banker and tradesmen. He believed that he was in harmony with all present in saying that the administration of the hospital was wise in the decision it had arrived at. He hoped that it would be rewarded for its action, which must have cost it much anxious thought. He sincerely trusted that the public, generally so warm and hearty in supporting hospitals, would come forward to help a charity which had done so much as this. (Cheers.) Another item in the expenditure of the hospital was that of re-drainage. Unfortunately, he had had some experience of drainage matters, both privately and officially, and he knew that it was most troublesome and expensive. But it was a matter that had to be faced, and the hospital, like everybody, must put its drainage in order. The hospital was administered on the most economical principles, and to ensure efficiency as well as economy was one of the greatest difficulties in the management of all public institutions. He might add that the hospital was rather different from many others in that it had not willingly come forward in the way of festivals. Eleven years had passed since one was held, and only necessity led to the present appeal being made. It had been his good fortune to preside on many such occasions, and he had always felt most grateful to those whom he had the honour of addressing for the support they had given him that evening. A good cause like that of a hospital was not generally pleaded for in vain. (Cheers.)

